# Managing cardiovascular risk factors with telemedicine in primary care: A systematic review and meta-analysis of patients with arterial hypertension and type 2 diabetes

**DOI:** 10.1177/17423953241277896

**Published:** 2024-08-28

**Authors:** Matic Mihevc, Tina Virtič Potočnik, Črt Zavrnik, Zalika Klemenc-Ketiš, Antonija Poplas Susič, Marija Petek Šter

**Affiliations:** 1 Primary Healthcare Research and Development Institute, 37676 Community Health Centre Ljubljana, Ljubljana, Slovenia; 2 Medical Faculty, Department of Family Medicine, 37664 University of Ljubljana, Ljubljana, Slovenia; 3 Medical Faculty, Department of Family Medicine, 54765 University of Maribor, Maribor, Slovenia

**Keywords:** Telemonitoring, teleconsultations, blood pressure, HbA1c, lipids, health education

## Abstract

**Objectives:**

To review the effect of telemedicine interventions on cardiovascular risk factors control in people with arterial hypertension (AH), type 2 diabetes (T2D), or both in primary care.

**Methods:**

We conducted a systematic review in February 2024 using PubMed/MEDLINE, Cochrane Library, and EMBASE databases. We included randomised controlled trials from 2010 onwards, lasting ≥3 months, comparing telemedicine to standard care for managing cardiovascular risk factors in adults with AH, T2D, or both.

**Results:**

Among 1803 records, 54 were included. Telemonitoring with teleconsultations showed the best outcomes. For AH, systolic blood pressure decreased by −5.63 mmHg (95% CI −9.13 to −2.13) at 6 months and −5.59 mmHg (95% CI −10.03 to −1.14) at 12 months compared to standard care. For T2D, HbA1c decreased by −0.45% (95% CI −0.90 to 0.00) at 6 months and −0.18% (95% CI −0.41 to 0.05) at 12 months compared to standard care. Blood glucose self-monitoring was as effective as telemonitoring for T2D at 6 months. The effect on diastolic blood pressure, low-density lipoprotein, triglycerides, and body mass index was non-significant.

**Discussion:**

Telemedicine offers short-term benefits but lacks long-term effectiveness. Optimal outcomes require a combined telemedicine approach, health education co-intervention, ≥12-month follow-up, and careful patient selection.

## Introduction

The rising prevalence of arterial hypertension (AH) and type 2 diabetes (T2D) presents significant challenges to global healthcare systems, particularly impacting cardiovascular disease-related mortality as preventative measures remain inadequate.^1,2^

Development of cardiovascular disease in individuals with AH and T2D can be influenced by a variety of risk factors, both modifiable and non-modifiable. Effective control of blood pressure (BP) and blood glucose (BG) is crucial. From a clinical standpoint, a 10-mmHg reduction in systolic BP (SBP), 5-mmHg reduction in diastolic BP (DBP), and a 0.5% drop in glycated haemoglobin (HbA1c) is associated with up to a 20% reduction in major cardiovascular events and considered clinically relevant.^3–5^

However, cardiovascular disease is not only associated with high BP or BG levels but also to shared risk factors such as high triglycerides (TG), high low-density lipoprotein (LDL), obesity (usually measured as body mass index, BMI), or chronic kidney disease. Dysfunction in one system can exacerbate problems in others, leading to negative health effects. Consequently, interventions that target one or two risk factors may also impact others in a reciprocal manner.^1,5–7^

Primary care plays a central role in the management of AH and T2D, as well as in preventing their risk factors and complications. It serves as the main point of contact for patients and leads chronic disease management through screening, health education, treatment initiation, self-management support, and ongoing counselling to ultimately provide comprehensive and patient-centred healthcare.^8–12^

Despite its central role, primary care faces growing challenges such as staff shortages, fragmentation of care, unequal access, and the increasing complexity of chronic disease management.^10,13^ To overcome these challenges, telemedicine has emerged as a promising solution. Telemedicine, as defined by the World Health Organisation (WHO), facilitates the delivery of healthcare services across distances using information and communication technologies, encompassing diagnosis, treatment, prevention, research, evaluation, and professional education.^11,14,15^

In the management of AH and T2D, telemedicine streamlines integrated care processes by enabling early disease detection, treatment decisions, self-monitoring, patient education, provider coordination, and adherence to clinical pathways.^16^ While the COVID-19 pandemic has accelerated the adoption of simpler telemedicine approaches such as teleconsultations^17^ or asynchronous telemedicine^13,18^ in primary care, the implementation of more complex measures like telemonitoring, mobile health or tele-education continues to be constrained by financial resources.^19–21^

For future scale-up of telemedicine in primary care, it is vital to gather evidence regarding its clinical effectiveness and determine which remote care models deliver the optimal outcomes for managing AH and T2D. As telemedicine services have evolved, different approaches have emerged, and each approach or their combination should be evaluated. Previous reviews^22–26^ have focused primarily on BP and BG control in patients with isolated AH or T2D, whereas the effect on other risk factors and in cases of comorbid AH and T2D should also be explored.

The aim of this systematic review is to examine how various telemedicine approaches impact changes in cardiovascular risk factors, including SBP, DBP, HbA1c, LDL, TG, and BMI, compared to standard care in people with AH, T2D or both in primary care settings.

## Methods

### Design

A systematic review with meta-analysis was conducted following the Preferred Reporting Items for Systematic reviews and Meta-Analyses (PRISMA) guidelines.^27^ The research protocol was registered in the PROSPERO international register of systematic reviews, with the registration number 227335.

### Research questions

The research questions were formed based on the PIO strategy (population, intervention, outcome) and were as follows:

1. Which telemedicine intervention is the most effective in improving control of SBP, DBP, HbA1c, LDL, TG or BMI compared to standard care in people with AH, T2D, or both in primary care?
2. What is the difference in effectiveness of commonly used telemedicine approaches (telemonitoring, self-monitoring, teleconsultation, tele-education) for the control of SBP and HbA1c, depending on the combination used and the patient group (AH, T2D, or both)?

### Search algorithm

In the first part, we systematically reviewed the existing literature in electronic databases, including PubMed/MEDLINE, Cochrane Library, and EMBASE, to retrieve relevant articles. The search strategy compromised using Medical Subject Headings for the concepts: “telemedicine” “hypertension”, “diabetes type 2”, “blood pressure”, “blood glucose”, “HbA1c”, “cholesterol”, “body mass index”, and “primary health care”. We employed the Boolean operators such as “AND”, “NOT”, and “OR” to construct a comprehensive search algorithm, as detailed in Appendix 1. The initial search was performed in November 2020, and an update search was conducted in February 2024. In March 2024, we extended our collections of studies by conducting a comprehensive hand search of the publications from international bodies and their websites, including WHO, International Diabetes Federation, American Diabetes Association, and Centres for Disease Control. Additionally, we reviewed leading journals that focus on telemedicine and primary care, including Lancet Digital Health, npj Digital Medicine, Journal of Medical Internet Research, JMIR mHealth and uHealth, Journal of Telemedicine and Telecare, British Journal of General Practice, Annals of Family Medicine, European Journal of General Practice, Primary Care Diabetes, and Diabetes Research and Clinical Practice. We limited our search to original scientific articles published in English between 2010 and 2024 to focus on recent and significant literature while ensuring an adequate corpus of work for thorough analysis.

### Study selection

To determine the eligibility of documents for further analysis, we applied the PICOTS criteria as outlined in Table 1. We included randomised controlled trials (RCTs) that assessed the effectiveness of telemedicine interventions in adults with AH, T2D or both in primary care setting. The decision to focus on RCTs was based on the research question and objectives of this systematic review. Studies were required to report a measurable change in at least one outcome of interest after a minimum follow-up period of 3 months, as this provides sufficient time to estimate intervention effects and stability of measurements and is consistent with methodology in previous reviews.^1,28^ While our review primarily centred on telemedicine interventions, we also considered synchronous patient-provider tele-education approaches. Originally encompassed within the broader category of telehealth,^14^ this term has been merged with other forms of telemedicine in several RCTs. Consequently, we deemed it important to examine its role independently to fully comprehend its effectiveness.

**Table 1.** PICOTS inclusion and exclusion criteria.

PICOTS	Inclusion criteria	Exclusion criteria
Population	▪ People with AH, T2D or both ▪ ≥ 18 years of age	▪ People with other conditions ▪ <18 years of age
Intervention	▪ Telemedicine approaches as defined by the WHO,^15^ including patient tele-education	▪ Health applications related to nutrition, diet, mental health, adherence, or education without involvement of a healthcare professional ▪ Telemedicine interventions using only artificial intelligence algorithms and no human interaction
Control	▪ Standard care involving face-to-face consultations	▪ Standard care supplemented with telemedicine or educational co-intervention
Outcome	▪ Change in SBP, DBP, HbA1c, TG, LDL, BMI	▪ Other outcomes
Timeframe	▪ A follow-up period ≥3 months	▪ A follow-up period < 3 months
Study design	▪ Randomised controlled design ▪ Primary care setting	▪ Qualitative study design ▪ Protocol for clinical study ▪ Hospital setting

### Data analysis

#### Qualitative extraction of data

Two independent researchers (MM & MPŠ) conducted screenings of the datasets using data extraction tool that included various study-related characteristics, including author, publication year, country, study design/setting, sample size, and inclusion criteria. Additionally, we extracted telemedicine intervention-related characteristics, such as the type and duration of the intervention, outcomes measured, and main findings. For categorising telemedicine approaches, we used definitions described in Table 2.

**Table 2.** Definition of approaches used in telemedicine interventions.

Approach	Definition
Telemonitoring	The use of validated automated devices that transmit patient vital signs and other data in real time to a monitoring centre, where abnormalities trigger a rapid response from health professionals.^11,14,15^
Self-monitoring	Monitoring vital signs at home using medical devices, without the need for an internet connection or real-time contact with healthcare providers. Users can enter readings into a health application for optional asynchronous feedback from health professionals.^29^
Mobile health (mHealth)	Practice of medicine supported by mobile devices and/or wireless infrastructure, often for the purpose of telemonitoring or tele-education.^30^
Teleconsultation	Medical consultation between patient and health professional synchronously via videoconferencing equipment or telephone without face-to-face contact.^14^
Tele-education	Distance health promotion and self-management education using information and communication technologies with the involvement of health professionals.^31^

#### Quantitative extraction of data

In the quantitative analysis, we examined the mean differences in SBP, DBP, HbA1c, LDL, TG, and BMI between telemedicine and standard care group over a period of 3 to 60 months. To ensure consistency, we standardised all results by converting them from their original units to the International System of Units commonly used in Europe. Specifically, for TG, we divided the results originally measured in milligrams per decilitre (mg/dL) by 88.57 to obtain values in millimoles per litre (mmol/L). Similarly, for LDL, we divided the results originally measured in mg/dL by 38.67 to obtain values in mmol/L.^32^

Next, we calculated the mean difference between groups (Δ_I−C_) to determine the variation in outcomes between the intervention and control groups. This involved subtracting the change from baseline to the observed timeframe in the intervention group (Δ_I_) from the corresponding change in the control group (Δ_C_). This process allowed us to assess the overall impact of the intervention compared to the control group. If Δ_I−C_ was already reported in the article, we extracted this data from the text along with the corresponding p-values.^33^

#### Assessment of risk of bias

Two independent researchers (MM & MPŠ) evaluated the quality of the included studies using Version 2 of the Cochrane tool for assessing bias in randomised trials.^34^ Disagreements were resolved by discussion, or, where this was not possible, by consultation with a research team (ČZ, TVP, ZKK, APS). The risk of bias assessment considered five domains: randomisation process, deviations from intended interventions, missing outcome data, outcome measurement, selection of reported results. Ultimately, all domains were categorised as having either a low risk, some concerns, or a high risk of bias.

### Meta-analysis

In meta-analysis, we aimed to evaluate the effectiveness of prevalent telemedicine approaches, focusing on interventions such as self-monitoring or telemonitoring of BP and BG, coupled with teleconsultations or tele-education, in comparison to standard care. Specifically, we examined their impact on two critical cardiovascular risk factors, SBP and HbA1c, over 6 and 12 months from baseline. Due to limited data availability, we were unable to conduct reliable meta-analyses for other telemedicine approaches or outcomes.

For our meta-analysis, we included only RCTs reporting means and standard deviations (SD) or 95% confidence intervals (CI) of observed outcomes. We followed the methods described in the Cochrane Handbook for Systematic Reviews of Interventions (version 6.4, 2023) for calculations. In cases where studies reported 95% CIs instead of SDs, we derived SDs using the formula: SD = √N×(upper limit – lower limit)/3.92, where N is the number of participants. For studies reporting standard errors (SE), SD was derived using the formula: SD = SE×√N, where N is the number of participants.

Next, we employed the inverse variance approach in Review Manager (RevMan) version 5.4.1 by The Cochrane Collaboration to calculate pooled mean differences and 95% CIs. Heterogeneity was assessed using I^2^ statistic, where a value below 50% indicated non-significant heterogeneity and prompted the use of a fixed-effect model, while values above 50% indicated significant heterogeneity, leading to the use of a random-effects model.^35^

Finally, we assessed publication bias by visual inspection of funnel plots. When more than 3 studies were included in the meta-analysis, we assessed publication bias quantitatively using Egger's regression test, which compares effect sizes with their precision (1/SE). Calculations were performed using IBM SPSS Statistics (version 25.0), with a p-value <0.05 indicating possible publication bias.^36^

### Certainty of the evidence

To determine the certainty of the evidence for the RCTs included in our meta-analysis, we used the Grading of Recommendations, Assessment, Development, and Evaluation (GRADE) tool. This tool systematically assesses several factors: risk of bias, inconsistency, indirectness, imprecision, and publication bias.^37^ Two independent researchers (MM & MPŠ) rated each section. The overall GRADE score, ranging from very low (⊕), low (⊕⊕), moderate (⊕⊕⊕) to high (⊕⊕⊕⊕), was determined by discussion. If no consensus could be reached between MM & MPŠ, we consulted a larger research team (ČZ, TVP, ZKK, APS).

## Results

### Search results

**Figure 1.**
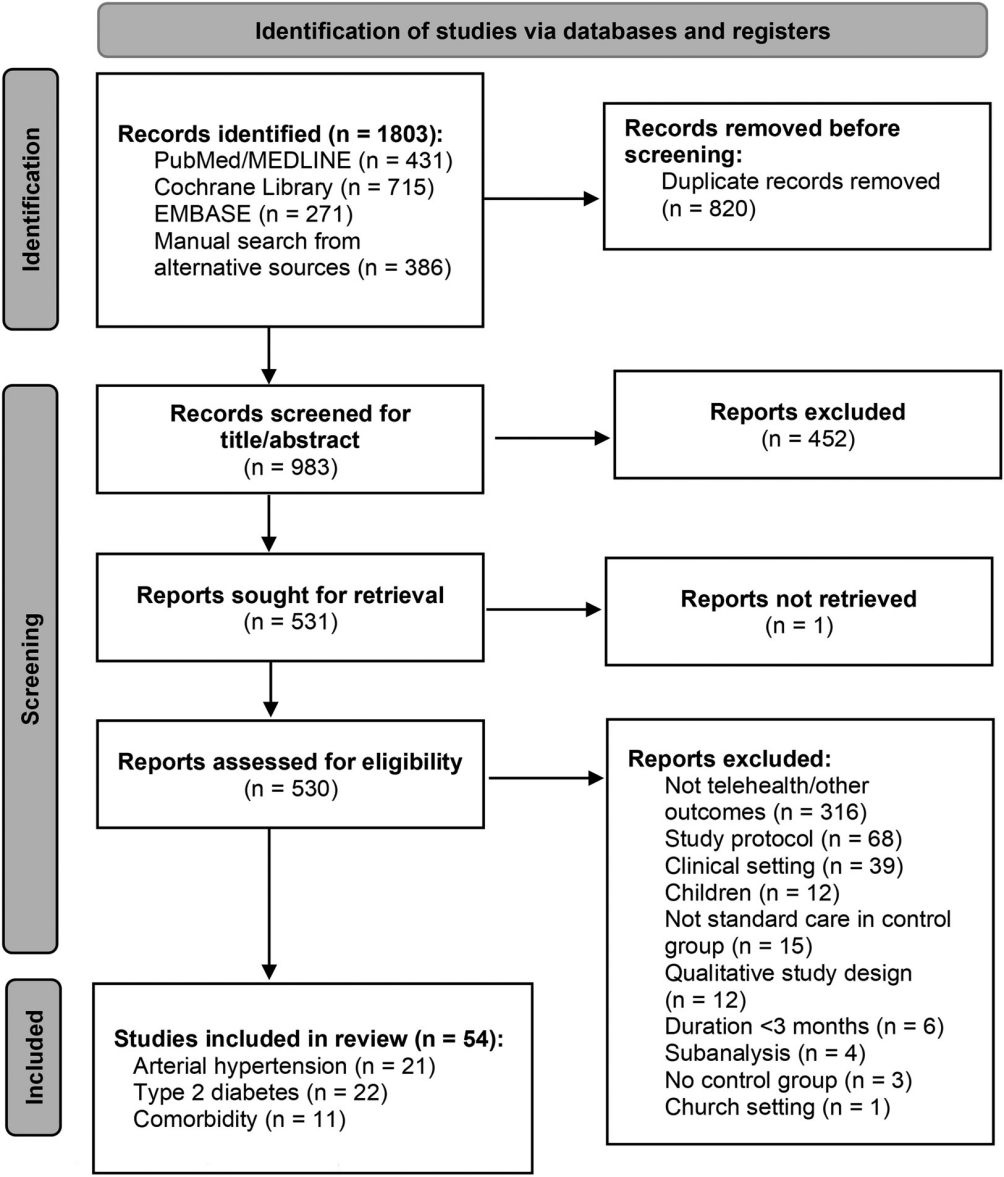
PRISMA flow diagram.

The search algorithm yielded a total of 1803 references, as shown in Figure 1. After eliminating duplicate entries and screening the titles and abstracts, 1273 references were excluded. Following a thorough evaluation of the full texts, an additional 476 references were excluded. Ultimately, the analysis included 54 references. Details on the risk of bias assessment of included studies are provided in Appendix 2. Overall, 25 exhibited a low risk of bias, 27 had some concerns, and 2 were identified as having a high risk of bias.

### Effect of telemedicine interventions on blood pressure

Telemedicine interventions have shown varying levels of effectiveness in controlling BP in people with AH, with only a limited number of interventions achieving a clinically significant reduction of 10 mmHg in SBP and 5 mmHg in DBP.^38,39^ Table 3 summarises the reported effects of different telemedicine interventions on cardiovascular risk factors, including changes in SBP and DBP in people with AH. Notably, interventions combining BP telemonitoring with teleconsultations have consistently shown the most prominent results.

**Table 3.** Reported effects of different telemedicine interventions on risk factors control in people with arterial hypertension. Results are presented as the mean difference ( Δ ) between telemedicine and standard care group, with statistically significant differences indicated by an asterisk (*).

Author (reference)	Sample size	Duration	Δ SBP [mmHg]	Δ DBP [mmHg]	Δ HbA1c [%]	Δ LDL [mmol/L]	Δ TG [mmol/L]	Δ BMI [kg/m^2^]	Overall risk of bias
**Adherence telemonitoring with pill bottle**									
Mehta et al.^40^	149	4 M	+0.4	/	/	/	/	/	Some concerns
**Blood pressure telemonitoring**									
McManus et al.^41^	527	12 M	−5.4*	−2.7*	/	/	/	/	Low
**Blood pressure telemonitoring combined with teleconsultations**									
Hoffmann Petersen et al.^42^	356	3 M	0.0	0.0	/	/	/	/	Some concerns
McKinstry et al.^43^	401	6 M	−4.3*	−2.3*	/	/	/	/	Low
Teo et al.^44^	217	6 M	−5.1	−4.4	/	/	/	/	Some concerns
Bove et al.^45^	241	6 M	−4.3	−2.2	/	−0.04	−0.18	−0.70	Some concerns
Logan et al.^46^	110	12 M	−6.8*	−3.6*	/	/	/	/	Some concerns
Margolis et al.^47^	3071	12 M	−0.8	+0.3	/	/	/	/	Low
McManus et al.^29^	1182	12 M	−4.7*	−1.3*	/	/	/	/	Low
Bosworth et al.^48^	296	18 M	−1.2	−0.5	/	/	/	/	Some concerns
Maciejewski et al.^49^	296	18 M (I) + 18 M (FU)	−3.6	/	/	/	/	/	Low
Margolis et al.^50^	450	12 M (I) + 42 M (FU)	−2.5	−1.0	/	/	/	/	Some concerns
**Blood pressure telemonitoring combined with tele-education**									
Bosworth et al.^48^	295	18 M	+2.2	+0.6	/	/	/	/	Some concerns
Maciejewski et al.^49^	295	36 M	−5.0*	/	/	/	/	/	Low
**Blood pressure telemonitoring combined with teleconsultations and tele-education**									
Wakefield et al.^51^	200	12 M	−7.5*	−3.5*	/	/	/	/	Some concerns
Bosworth et al.^48^	294	18 M	−3.6	−1.4	/	/	/	/	Some concerns
Maciejewski et al.^49^	294	18 M (I) + 18 M (FU)	−5.5*	/	/	/	/	/	Low
**Blood pressure self-monitoring combined with teleconsultations**									
McManus et al.^29^	1182	12 M	−3.5*	−1.5*	/	/	/	/	Low
Bray et al.^52^	263	12 M	−5.5	−1.0	/	/	/	/	Low
Kerry et al.^53^	381	12 M	+0.3	/	/	/	/	/	Low
Fu et al.^39^	420	18 M	−10.4*	−6.4*	/	−0.02	/	−0.28	Some concerns
**Blood pressure self-monitoring combined with teleconsultations and health education**									
McManus et al.^41^	622	12 M	−3.5	−1.0	/	/	/	/	Low
**mHealth tele-education**									
Vedanthan et al.^54^	960	15 M	−3.4	+1.4	/	/	/	/	Some concerns
Marquez Contreras et al.^55^	148	12 M	−2.4	−2.6	/	/	/	/	Some concerns
**Virtual visits**									
Levine et al.^56^	1051	6 M	+0.4	/	/	/	/	/	Some concerns

**Legend:** SBP, systolic blood pressure; DBP, diastolic blood pressure; HbA1c, glycated haemoglobin; LDL, low-density lipoprotein; TG, triglycerides; BMI, body mass index; M, month; I, intervention; FU, follow-up.

In the meta-analyses examining the effect of telemedicine interventions on SBP change compared to standard care (Table 4), the combination of BP telemonitoring with teleconsultations significantly reduces SBP at both 6- and 12-month intervals in patients with AH, exceeding the 5-mmHg threshold. However, when considering patients with AH and T2D, the impact is irrelevant, with minimal changes in SBP observed. The effect of BP self-monitoring combined with teleconsultation on SBP was insignificant. There was a high level of heterogeneity between studies, coupled with low publication bias. Certainty of the evidence was low to moderate. More detailed data are presented in Appendix 3.

**Table 4.** Effect of telemedicine interventions on the change in systolic blood pressure (mmHg) compared to standard care, depending on the observed population, intervention type, and duration.

Population	Duration	Trials	References	Patients, n	MD in SBP (95% CI)	p	I^2^ (%)	Egger's test (t value, p)	Certainty of the evidence (GRADE)
**BP self-monitoring combined with teleconsultations**									
AH	6 M	3	^29,39,53^	1333	−3.20 mmHg (−9.54, 3.13)	0.320	88	t = 1.540, p = 0.367	⊕ ⊕
AH	12 M	4	^29,39,52,53^	1546	−4.26 mmHg (−9.93, 1.40)	0.140	86	t = 0.448, p = 0.698	⊕ ⊕
**BP telemonitoring combined with teleconsultations**									
AH	6 M	4	^29,38,43,45^	1741	−5.63 mmHg (−9.13, −2.13)	0.002	74	t = 0.034, p = 0.976	⊕ ⊕ ⊕
AH and T2D	6 M	2	^57,58^	439	+0.22 mmHg (−1.94, 2.39)	0.840	13	NA	⊕ ⊕
AH	12 M	4	^29,38,46,47^	4248	−5.59 mmHg (−10.03, −1.14)	0.010	86	t = 3.076, p = 0.091	⊕ ⊕ ⊕
AH and T2D	12 M	2	^57,59^	510	+0.31 mmHg (−1.69, 2.30)	0.760	25	NA	⊕ ⊕ ⊕

**Legend**: BP, blood pressure; AH, arterial hypertension; T2D, type 2 diabetes; MD, mean difference; SBP, systolic blood pressure; 95% CI, 95% confidence interval; I^2^, heterogeneity index; NA, not applicable; GRADE, Grading of Recommendations, Assessment, Development, and Evaluation.

In terms of DBP control, combining BP telemonitoring with teleconsultations in patients with AH resulted in mean reductions in DBP ranging from −2.2 to −4.4 mmHg after 6 months^43–45^ and from −3.6 to +0.3 mmHg after 12 months.^29,38,47^ Isolated interventions, such as teleconsultations or adherence monitoring,^40,53^ virtual visits,^56^ or isolated mHealth tele-education approaches^54,55^ were found to be ineffective. A similar lack of clinical impact has been observed in patients with comorbid AH and T2D.^60–59^

### Effect of telemedicine interventions on glycaemic control

The effectiveness of telemedicine interventions in controlling BG levels in people with isolated T2D or comorbid AH and T2D varies with the number of telemedicine approaches combined, with multicomponent interventions achieving a clinically significant mean reduction of 0.5% in HbA1c after 6 months.^62–65^ However, this effect was not maintained beyond 12 months.^62,63,66–70^ A detailed overview of the reported effects of different telemedicine interventions on cardiovascular risk factors, including changes in HbA1c, in people with T2D or comorbid AH and T2D is shown in Table 5 and Table 6, respectively.

**Table 5.** Reported effects of different telemedicine interventions on risk factors control in people with type 2 diabetes. Results are presented as the mean difference ( Δ ) between telemedicine and standard care group, with statistically significant differences indicated by an asterisk (*).

Author (reference)	Sample size	Duration	Δ SBP [mmHg]	Δ DBP [mmHg]	Δ HbA1c [%]	Δ LDL [mmol/L]	Δ TG [mmol/L]	Δ BMI [kg/m^2^]	Overall risk of bias
**Blood glucose self-monitoring**									
Parsons et al.^71^	298	12 M	/	/	−0.75*	/	/	−0.28	Low
**Blood glucose self-monitoring combined with teleconsultations**									
Fortmann et al.^62^	126	6 M	+3.8	+1.0	−0.80*	−0.01	−0.09	+0.50	Some concerns
Parsons et al.^71^	299	12 M	/	/	−0.87*	/	/	+0.09	Some concerns
**Face-to-face training with pharmacist combined with teleconsultations**									
Lum et al.^72^	264	6 M	−13.5	/	−0.30*	−0.30	−0.20	/	Some concerns
**Healthcare workers video tele-education and teleconsultations**									
Naik et al.^73^	225	12 M	/	/	−0.06	/	/	/	Some concerns
Leong et al.^68^	181	3 M	/	/	+0.06	/	/	/	Low
**Health coach tele-education**									
Christensen et al.^65^	170	6 M	+1.4	−0.7	−0.15	−0.17	−0.41	−0.89*	Low
**mHealth pharmacist teleconsultation and tele-education**									
Gerber et al.^74^	221	12 M	−3.1	−2.4	−0.62*	+0.07	+0.09	−1.25	Low
**Blood glucose self-monitoring thought mHealth or other means combined with teleconsultations and tele-education**									
Odnoletkova et al.^75^	574	6 M (I) + 12 M (FU)	−2.0	0	−0.20*	−0.07	−0.17	−0.40	Some concerns
Liou et al.^76^	95	6 M	−5.7	+0.4	−0.60*	+0.19	0.00	−1.00	Some concerns
Vaughan et al.^64^	89	6 M	−6.9*	−3.2	−0.98*	/	/	−0.84	Some concerns
Lim et al.^77^	204	6 M	−2.8	−2.4	−0.40*	−0.09	+0.11	↓	Low
Iljaž et al.^67^	107	12 M	−2.8	+0.5	−0.60*	+0.10	0.00	−0.60	Low
**Blood glucose +/- blood pressure telemonitoring combined with teleconsultations**									
Yang et al.^78^	125	3 M	−3.7	−2.7	−0.35	−0.08	0.00	+0.15	Low
Warren et al.^63^	157	6 M	−12.0	−3.0	−0.90*	/	/	+0.80	Some concerns
Bujnowska-Fedak et al.^79^	100	6 M	+2.0	−2.5	−0.08	/	/	−0.80	Some concerns
Mudiyanselage et al.^80^	177	12 M	−1.5	+1.42	−0.22	−0.02	−0.04	/	Some concerns
Steventon et al.^69^	457	12 M	/	/	−0.21*	/	/	/	Low
McFarland et al.^81^	110	6 M	/	/	−0.50	/	/	/	Some concerns
Tang et al.^66^	415	12 M	−2.3	−0.7	−0.19	−0.16*	/	0.00	Low
Lee et al.^70^	240	12 M	−0.6	−0.6	−0.03	+0.01	+0.03	/	Some concerns
Jia et al.^82^	19 546	12 M	−0.8	−0.6	−0.32	**/**	−0.01	/	Low
**Blood glucose +/- blood pressure telemonitoring combined with teleconsultations and peer support**									
Anzaldo Campos et al.^83^	301	10 M	−3.4	−2.0	−1.70*	−0.02	−0.24	+0.31	Some concerns

**Legend:** SBP, systolic blood pressure; DBP, diastolic blood pressure; HbA1c, glycated haemoglobin; LDL, low-density lipoprotein; TG, triglycerides; BMI, body mass index; M, month; I, intervention; FU, follow-up.

**Table 6.** Reported effects of different telemedicine interventions on risk factors control in people with arterial hypertension and type 2 diabetes. Results are presented as the mean difference ( Δ ) between telemedicine and standard care group, with statistically significant differences indicated by an asterisk (*).

Author (reference)	Sample size	Duration	Δ SBP [mmHg]	Δ DBP [mmHg]	Δ HbA1c [%]	Δ LDL [mmol/L]	Δ TG [mmol/L]	Δ BMI [kg/m^2^]	Overall risk of bias
**Diabetologist teleconsultations**									
Basudev et al.^84^	208	12 M	−8.0*	−2.0*	+0.20	/	/	−0.07	Some concerns
**Nutritionist teleconsultations and tele-education**									
Benson et al.^85^	118	12 M	/	/	−0.21	−0.27	/	+2.19	Low
**Adherence telemonitoring with pillbox combined with pharmacist teleconsultations**									
Choudhry et al.^86^	4078	12 M	+2.3	/	+0.20	−0.12	/	/	Low
**Telemonitoring with pill ingestible sensor and body patch**									
Frias et al.^87^	109	3 M	−4.6	−2.4	−0.48	−0.28	/	/	High
**Blood glucose self-monitoring combined with teleconsultations and tele-education**									
Ramallo-Farina et al.^60^	1123	24 M	−1.49	−0.82	−0.15	+0.05	−0.07	−0.20	Low
Weinstock et al.^61^	1665	60 M	−4.3*	−2.6*	−0.29*	−0.10	/	/	Low
**Blood glucose +/- blood pressure telemonitoring combined with teleconsultations**									
Stone et al.^58^	150	6 M	−1.0	−3.5	−0.70*	−0.22	−0.20	↑	Low
Wild et al.^88^	321	9 M	−3.1*	−2.2*	−0.51*	/	/	↓	Low
Nicolucci et al.^57^	302	12 M	+0.7	+0.4	−0.33*	−0.02	−0.15	↑	Some concerns
Karhula et al.^59^	250	12 M	−0.2	−0.7	−0.11	+0.04	+0.07	↑	Low
Wakefield et al.^89^	209	12 M	−8.2	/	+0.14	/	/	/	High

**Legend:** SBP, systolic blood pressure; DBP, diastolic blood pressure; HbA1c, glycated haemoglobin; LDL, low-density lipoprotein; TG, triglycerides; BMI, body mass index; M, month; I, intervention; FU, follow-up.

In the meta-analyses (Table 7) examining the effect of telemedicine interventions on HbA1c change compared to standard care, the combination of two interventions (BG telemonitoring and teleconsultations) or three interventions (BG self-monitoring, teleconsultations, and tele-education) resulted in a mean reduction in HbA1c of around 0.5%. Notably, the effect was more consistent with the combination of three interventions. In contrast, the effect of the combination of teleconsultations and tele-education was clinically and statistically insignificant. Furthermore, the reduction in HbA1c at 12 months did not meet the clinically significant threshold of 0.5% for any of the interventions. Notably, the reduction observed in trials combining BG telemonitoring and teleconsultations remains statistically significant. The heterogeneity between RCTs was high for those involving BG telemonitoring but low for those involving BG self-monitoring and/or tele-education. Publication bias was low, except for interventions involving BG self-monitoring. Certainty of the evidence was low to moderate. More detailed data are provided in Appendix 3.

**Table 7.** Effect of telemedicine interventions on the change in HbA1c (%) compared to standard care, depending on the observed population, intervention type, and duration.

Population	Duration	Trials	References	Patients, n	MD in HbA1c (95% CI)	p	I^2^ (%)	Egger's test (t value, p)	Certainty of the evidence (GRADE)
**BG self-monitoring combined with teleconsultations and tele-education**									
T2D	6 M	7	^62,64,67,71,75–77^	1639	−0.47% (−0.60, −0.35)	<0.001	44	t = 3.061, p = 0.028	⊕ ⊕ ⊕
**BG telemonitoring combined with teleconsultations**									
T2D	6 M	5	^63,66,70,79,81^	887	−0.45% (−0.90, 0.00)	0.050	87	t = 1.051, p = 0.370	⊕ ⊕ ⊕
T2D and AH	6 M	2	^57,58^	439	−0.53% (−1.07, 0.00)	0.050	61	NA	⊕ ⊕
T2D	12 M	4	^66,69,70,82^	18,558	−0.18% (−0.41, 0.05)	0.120	95	t = 0.231, p = 0.839	⊕ ⊕ ⊕
T2D and AH	12 M	2	^57,59^	519	−0.21% (−0.37, −0.06)	0.008	0	NA	⊕ ⊕ ⊕
**Teleconsultations combined with tele-education**									
T2D	6 M	4	^65,72–74^	709	−0.14% (−0.38, 0.10)	0.240	45	t = −0.420, p = 0.715	⊕ ⊕ ⊕
T2D	12 M	2	^73,74^	384	−0.36% (−0.83, 0.10)	0.130	17	NA	⊕ ⊕

**Legend**: BG, blood glucose; AH, arterial hypertension; T2D, type 2 diabetes; MD, mean difference; HbA1c, glycated haemoglobin; 95% CI, 95% confidence interval; I^2^, heterogeneity index; NA, not applicable; GRADE, Grading of Recommendations, Assessment, Development, and Evaluation.

### Effect of telemedicine interventions on lipid profile

The effect of telemedicine interventions on lipid profile control in people with AH and/or T2D is clinically irrelevant. In people with T2D the mean change in LDL is ranging from −0.30 to +0.10 mmol/L, whereas the mean change in TG is ranging from −0.41 to +0.11 mmol/L.^62,65,67,72,74,75,77,82^ In people with comorbid AH and T2D the mean change in LDL is ranging from −0.28 to +0.05 mmol/L, whereas the mean change in TG is ranging from −0.20 to +0.07 mmol/L.^58,60–59,85–87^ As the evidence is inconsistent, it is not possible to stratify effectiveness results by intervention type or duration. In both groups, the effectiveness was greater in intervention that included tele-education.^65,75,85^

### Effect of telemedicine interventions on body mass index

The effect of telemedicine interventions on BMI in people with AH and/or T2D is clinically irrelevant. In people with T2D the mean change in BMI ranges from −1.25 to +0.80 kg/m^2^,^62–67,71,74,75,78,79,83^ whereas in people with AH and T2D the mean change in BMI ranges from −0.20 to +2.19 kg/m^2^.^60,84,85^ As the evidence is inconsistent, it is not possible to stratify effectiveness results by intervention type or duration.

## Discussion

### Summary of findings and comparison with the existing literature

The systematic review indicates significant improvements in SBP and HbA1c across various telemedicine approaches for both AH and T2D, while the impact on DBP, lipid profile and BMI was clinically insignificant. However, studies consistently show stronger evidence of positive outcomes in people with T2D compared to those with AH. Positive outcomes are demonstrated as early as 3 months, with more consistent and sustained improvements seen with longer durations, particularly over 12 months. However, the effectiveness and ideal duration vary depending on the type of intervention and patient group.

In contrast to previous reviews that mainly focused on the impact of telemedicine approaches on BP and BG control in primary care,^22,23^ or specifically examined a single type of telemedicine, such as synchronous telemedicine,^24^ or limited their analysis to people with AH^25^ or T2D,^22,23,26^ our review encompasses the entire spectrum of telemedicine approaches for people with AH, T2D, or both.

The most effective method of managing AH entails a combination of BP telemonitoring and teleconsultations.^29,38^ Our meta-analysis found that this approach exceeded the 5-mmHg threshold but fell short of the desired 10-mmHg SBP target. Long-term benefits have been observed with BP telemonitoring interventions lasting at least 12 months, extending up to 54 months, with the most significant impact seen within the first 18 months.^49,50^ These findings align with previous reviews, which highlighted specific subgroups benefiting more from telemedicine interventions, including individuals with higher BP, obesity, previous stroke, diabetes, blacks, low-income individuals, and those with low treatment adherence.^53,89–91^ In our meta-analysis, BP telemonitoring was effective only in patients with isolated AH and not in those with comorbid T2D. This may be due to study criteria focusing on HbA1c rather than changes in SBP, the emergence of novel T2D treatments that have a positive impact on BP control, and lower patient adherence to monitoring due to tracking multiple parameters.^11,21^ Further research is needed to better understand this phenomenon.

Several studies have shown a 0.5% reduction in HbA1c in people with T2D after 6 months,^62–65^ although this reduction tends to decrease after 12 months compared to standard care.^63,66,69,80^ Effective management of T2D in our meta-analysis involved either a combination of two approaches (BG telemonitoring and teleconsultations)^63,69,82^ or three approaches (BG self-monitoring, teleconsultations, and tele-education).^62,71^ BG telemonitoring had a stronger effect in individuals with AH and T2D compared to those with isolated T2D. Positive long-term effects were found when BG self-monitoring was combined with nurse-led telecoaching over 6 months, extending to 18 months from baseline.^75^ In another study, BG self-monitoring combined with teleconsultations and tele-education over a 5-year period maintained the positive effect over the entire intervention period.^61^ These findings align with previous reviews, which highlighted greater benefits for recently diagnosed T2D patients, those receiving telemonitoring or remote case management, and those with baseline HbA1c levels >8%.^28,92^

Despite the favourable impact of telemedicine on SBP and HbA1c levels, the current evidence suggests that its effect on changes in DBP, lipid profile and BMI is considered clinically irrelevant for both AH and T2D^58,60–59,85–87^ and is consistent with previous reviews.^28,92,93^ While some reviews described statistically significant reductions in BMI or LDL using telemedicine,^92,93^ no meta-analysis found a clinically significant reduction in BMI of 2.0 points or a reduction in LDL of 1.0 mmol/L, which would reduce the risk of cardiovascular events by 20%.^94^ However, the results were greater for BMI than for LDL reduction and were more pronounced when mHealth, video consultations or remote case management were used and when interventions lasted ≥6 months.^64,65,74,75,92^ To address risk factors more effectively, it is crucial to develop alternative interventions focusing on diet, physical activity, and health education. These could include structured lifestyle programmes, personalised dietary advice and tailored physical activity plans. Community-based initiatives, support groups or two-way SMS reminders could improve adherence.^8,16,95^ Furthermore, technology-based tools such as fitness trackers and diet monitoring apps can provide ongoing support and encourage positive health behaviours.^96^

### Implications for research and practice

Scaling up clinically effective telemedicine interventions in primary care requires consideration of patient-specific factors, including underlying conditions and expectations, health literacy, communication preferences, and complexity of system design.^12,13,16,21^ Additionally, specific inclusion criteria should be established for these interventions, with individuals having multimorbidity, older age, low treatment adherence, or recently diagnosed AH or T2D being ideal candidates.^28,97^ Proficiency in using modern technologies during pre-testing may also enhance the benefits of telemedicine interventions compared to less proficient users.^16^

Regarding the duration of telemedicine interventions, our meta-analysis highlights that the effectiveness, particularly in reducing SBP and HbA1c, is most notable within the initial 6 months but declines afterward. However, interventions lasting 6 to 12 months have shown favourable outcomes for up to 54 months from baseline.^49,50,61^

System design is crucial, with a combination of telemonitoring or mHealth with teleconsultations being recommended as the gold standard.^23,24,28,92,97^ Additionally, integrating health education into interventions can enhance their effectiveness and address lifestyle risk factors, potentially improving lipid profile and body weight.^49,60,64,65^

Despite the effectiveness of telemedicine interventions, our systematic review identified several limitations in the study design (lack of blinding of patients, increased risk of selection bias, high or unclear loss to follow-up), heterogeneity between studies, small sample sizes, and underreporting of relevant information. Future research should prioritise long-term studies with at least 5 years of follow-up, especially in the multimorbid elderly population.^11,13,97,98^ Furthermore, the incorporation of cardiovascular disease biomarkers such as HF1, HF2 or CKD273 into telemedicine interventions could further motivate patients to manage their conditions effectively.^99^

### Limitations

Although the study used a double-blind review method following PRISMA standards, it is important to acknowledge its limitations. Firstly, relevant studies may have been missed if authors did not use precise search terms or describe the primary care setting accurately. Secondly, some full-text articles were excluded because they did not meet the specific WHO definition of telemedicine used in the study, particularly in cases where there was no direct interaction with healthcare workers. Thirdly, due to the broad scope of the review, meta-analyses were only conducted for the most prevalent telemedicine approaches and outcomes reported. However, further research on less common approaches and their combinations beyond primary care should be conducted to obtain enough studies to power meta-analyses. Next, subjective judgements may have influenced the assessment of bias in included studies using the Cochrane tool, potentially leading to inconsistencies. However, we addressed this limitation with discussion among the broader research group. Finally, we used Egger's regression method to assess publication bias, whereas assessing publication bias in the small number of studies included in the meta-analysis is challenging and should be interpreted with caution.

## Conclusions

Telemedicine has emerged as a promising modality within primary care, with considerable potential to improve clinical outcomes in people with AH and T2D. Our review shows that the integration of telemedicine interventions is effective for up to 18 months in people with AH and up to five years in people with T2D. However, it is noteworthy that clinically significant benefits were mainly observed in people with T2D, especially within the initial six months, while the sustained reduction in HbA1c levels decreased over time. However, in the context of the expansion of telemedicine, it is notable that almost no telemedicine approach worsened control of AH and T2D compared with standard care, and in most cases, at least maintained a steady state. To ensure the optimal development of telemedicine in primary care, the establishment of evidence-based clinical guidelines with precise delineation of intervention modalities, target patient cohorts, and recommended intervention durations is imperative. This approach will not only facilitate the generation of robust scientific evidence but also promote clinically impactful and cost-effective outcomes.

## Supplemental Material

sj-docx-1-chi-10.1177_17423953241277896 - Supplemental material for Managing cardiovascular risk factors with telemedicine in primary care: A systematic review and meta-analysis of patients with arterial hypertension and type 2 diabetes Supplemental material, sj-docx-1-chi-10.1177_17423953241277896 for Managing cardiovascular risk factors with telemedicine in primary care: A systematic review and meta-analysis of patients with arterial hypertension and type 2 diabetes by Matic Mihevc, Tina Virtič Potočnik, Črt Zavrnik, Zalika Klemenc-Ketiš, Antonija Poplas Susič and Marija Petek Šter in Chronic Illness
