# Supplementary material for: Managing cardiovascular risk factors with telemedicine in primary care: A systematic review and meta-analysis of patients with arterial hypertension and type 2 diabetes
Source: Chronic Illn. 2024 Aug 28;21(1):3–24. doi: 10.1177/17423953241277896 (PMC11969891; doi:10.1177/17423953241277896)
Supplement: sj-docx-1-chi-10.1177_17423953241277896 - Supplemental material for Managing cardiovascular risk factors with telemedicine in primary care: A systematic review and meta-analysis of patients with arterial hypertension and type 2 diabetes [file sj-docx-1-chi-10.1177_17423953241277896.docx]

**Appendix 1: PUBMED/MEDLINE literature search strategy**

((((((((((("Primary Health Care"[Mesh]) OR ("Primary Health Care*"[tiab])) OR ("Primary Healthcare*"[tiab])) OR ("Primary Care*"[tiab])) OR ("Community Based Care*"[tiab])) OR ("Non Clinical Care*"[tiab])) OR ("Nonclinical Care*"[tiab])) OR ((("Family Practice"[Mesh]) OR ("Family Practice*"[tiab])) OR ("Family Medicine"[tiab]))) OR (("General Practice"[Mesh]) OR ("General Practice*"[tiab]))) AND ((((("Hypertension"[Mesh]) OR ("Hypertension*"[tiab])) OR ("High Blood Pressure*"[tiab])) OR (("Diabetes Mellitus"[Mesh]) OR ("Diabetes Mellitus"[tiab]))) OR (("Hyperglycemia"[Mesh]) OR ("Hyperglycemia*"[tiab])))) AND ((((("Telemetry"[Mesh]) OR ("Telemetry"[tiab])) OR ("Telemetries"[tiab])) OR (((((((((("Telemedicine"[Mesh]) OR ("Telemedicine"[tiab])) OR ("mHealth"[tiab])) OR ("Telehealth"[tiab])) OR ("eHealth"[tiab])) OR ("Remote Monitoring"[tiab])) OR ("Remote Control*"[tiab])) OR ("Remote Tracking*"[tiab])) OR ("Telemonitoring*"[tiab])) OR ("Telemanagement"[tiab]))) OR (("Telecommunications"[Mesh]) OR ("Telecommunication*"[tiab])))) AND (((((((((((((((((((((((("Blood Pressure"[Mesh]) OR ("Blood Pressure*"[tiab])) OR ("Diastolic Pressure*"[tiab])) OR ("Systolic Pressure*"[tiab])) OR ("Pulse Pressure*"[tiab])) OR (((("Blood Glucose"[Mesh]) OR ("Blood Glucose*"[tiab])) OR ("Glucose Level*"[tiab])) OR ("Blood Sugar*"[tiab]))) OR ((((((((("Glycated Hemoglobin A"[Mesh]) OR ("Glycated Hemoglobin A"[tiab])) OR ("Hb A1a+b"[tiab])) OR ("HbA1c"[tiab])) OR ("Glycohemoglobin A"[tiab])) OR ("Hemoglobin A(1)"[tiab])) OR ("Hb A1b"[tiab])) OR ("Glycosylated A1b Hemoglobin"[tiab])) OR ("Glycosylated Hemoglobin*"[tiab]))) OR ((("Cholesterol"[Mesh]) OR ("Cholesterol*"[tiab])) OR ("Epicholesterol*"[tiab]))) OR ((("Dyslipidemias"[Mesh]) OR ("Dyslipidemia*"[tiab])) OR ("Dyslipoproteinemia*"[tiab]))) OR ((((("Hypercholesterolemia"[Mesh]) OR ("Hypercholesterolemia*"[tiab])) OR ("High Cholesterol Level*"[tiab])) OR ("Elevated Cholesterol*"[tiab])) OR ("Hypercholesteremia*"[tiab]))) OR (((("Hyperlipidemias"[Mesh]) OR ("Hyperlipidemia*"[tiab])) OR ("Lipidemia*"[tiab])) OR ("Lipemia*"[tiab]))) OR ((((("Body Mass Index"[Mesh]) OR (("Body Mass Index*"[tiab]))) OR ("Quetelet Index*"[tiab])) OR ("Quetelet's Index*"[tiab])) OR ("Quetelets Index*"[tiab]))) OR (("Body Weight"[Mesh]) OR ("Body Weight*"[tiab]))) OR (((("Obesity"[Mesh]) OR ("Obesities"[tiab])) OR ("Obesity"[tiab])) OR ("Bariatrics"[tiab]))) OR (("Overweight"[Mesh]) OR ("Overweight*"[tiab]))) OR ((((((("Metabolic Syndrome"[Mesh]) OR ("Metabolic Syndrome*"[tiab])) OR ("Insulin Resistance Syndrome*"[tiab])) OR ("Metabolic X Syndrome*"[tiab])) OR ("Reaven Syndrome X"[tiab])) OR ("Metabolic Cardiovascular Syndrome*"[tiab])) OR ("Cardiometabolic Syndrome*"[tiab])))

**APPENDIX 2: RISK OF BIAS ASSESSMENT**

**Table 1.** Risk of bias assessment for randomised controlled trials in patients with arterial hypertension

| **Author (reference)** | **Random sequence generation** | **Deviations from the intended interventions** | **Missing outcome data** | **Measurement of the outcome** | **Selection of the reported results** | **Overall risk of bias** |
| --- | --- | --- | --- | --- | --- | --- |
| Mehta et al. (1) | + | + | ? | ? | + | **?** |
| McManus et al. (2) | + | + | + | + | + | **+** |
| Hoffmann Petersen et al. (3) | + | + | ? | ? | + | **?** |
| McKinstry et al. (4) | + | ? | + | + | + | **+** |
| Bove et al. (5) | + | ? | ? | ? | + | **?** |
| Margolis et al. (6) | + | ? | - | ? | ? | **?** |
| McManus et al. (7) | + | ? | + | + | + | **+** |
| Bosworth et al. (8) | + | + | ? | ? | + | **?** |
| Maciejewski et al. (9) | + | ? | + | + | + | **+** |
| Margolis et al. (10) | + | ? | ? | ? | + | **?** |
| Wakefield et al. (11) | + | + | ? | ? | + | **?** |
| Logan et al. (12) | + | + | ? | ? | + | **?** |
| Bray et al. (13) | + | ? | + | + | + | **+** |
| Kerry et al. (14) | + | + | + | + | + | **+** |
| Fu et al. (15) | + | ? | + | ? | + | **?** |
| McManus et al. (16) | + | + | + | + | + | **+** |
| Vedanthan et al. (17) | + | + | + | ? | ? | **?** |
| Marquez Contreras et al. (18) | + | + | + | ? | ? | **?** |
| Margolis et al. (19) | + | + | + | ? | + | **+** |
| Teo et al. (20) | + | + | + | ? | ? | **?** |
| Levine et al. (21) | + | + | ? | ? | + | **?** |

**Legend**: + = Low risk, - = High risk, ? = Some concerns

**Table 2**. Risk of bias assessment for randomised controlled trials in patients with type 2 diabetes

| **Author (reference)** | **Random sequence generation** | **Deviations from the intended interventions** | **Missing outcome data** | **Measurement of the outcome** | **Selection of the reported results** | **Overall risk of bias** |
| --- | --- | --- | --- | --- | --- | --- |
| Iljaž et al. (22) | + | + | ? | + | + | **+** |
| Parsons et al. (23) | + | ? | + | + | + | **+** |
| Fortmann et al. (24) | + | + | ? | ? | + | **?** |
| Naik et al. (25) | + | ? | ? | ? | + | **?** |
| Odnoletkova et al. (26) | + | ? | ? | ? | + | **?** |
| Yang et al. (27) | + | + | + | ? | + | **+** |
| Warren et al. (28) | + | ? | ? | ? | ? | **?** |
| Bujnowska-Fedak et al. (29) | + | ? | ? | ? | + | **?** |
| Lim et al. (30) | + | + | + | ? | + | **+** |
| Liou et al. (31) | + | + | + | ? | ? | **?** |
| Mudiyanselage et al. (32) | + | ? | ? | ? | ? | **?** |
| Steventon et al. (33) | + | + | + | ? | + | **+** |
| Tang et al. (34) | + | + | + | + | + | **+** |
| McFarland et al. (35) | + | + | ? | ? | ? | **?** |
| Lee et al. (36) | + | ? | + | ? | + | **?** |
| Jia et al. (37) | + | + | ? | + | + | **+** |
| Anzaldo Campos et al. (38) | + | - | + | + | + | **?** |
| Vaughan et al. (39) | + | ? | + | ? | + | **?** |
| Reffstrup Christensen et al. (40) | + | + | ? | + | + | **+** |
| Lum et al. (41) | + | + | ? | ? | + | **?** |
| Gerber et al. (42) | + | + | + | + | + | **+** |
| Leong et al. (43) | + | + | + | + | + | **+** |

**Legend**: + = Low risk, - = High risk, ? = Some concerns

**Table 3**. Risk of bias assessment for randomised controlled trials in patients with arterial hypertension and type 2 diabetes

| **Author (reference)** | **Random sequence generation** | **Deviations from the intended interventions** | **Missing outcome data** | **Measurement of the outcome** | **Selection of the reported results** | **Overall risk of bias** |
| --- | --- | --- | --- | --- | --- | --- |
| Basudev et al. (44) | + | ? | ? | ? | + | **?** |
| Benson et al. (45) | + | ? | + | + | + | **+** |
| Choudhry et al. (46) | + | + | ? | + | + | **+** |
| Frias et al. (47) | + | ? | - | ? | - | **-** |
| Ramallo-Farina et al. (48) | + | + | ? | + | + | **+** |
| Stone et al. (49) | + | ? | + | + | + | **+** |
| Wild et al. (50) | + | ? | + | + | + | **+** |
| Nicolucci et al. (51) | + | ? | ? | + | + | **?** |
| Karhula et al. (52) | + | + | + | + | + | **+** |
| Wakefield et al. (53) | + | ? | - | ? | - | **-** |
| Weinstock et al. (54) | + | + | + | + | + | **+** |

**Legend**: + = Low risk, - = High risk, ? = Some concerns

**REFERENCES**

1. Mehta SJ, Volpp KG, Troxel AB, et al. Electronic pill bottles or bidirectional text messaging to improve hypertension medication adherence (Way 2 Text): a randomized clinical trial. *J Gen Intern Med* 2019; 34:2397-2404.
2. McManus RJ, Mant J, Bray EP, et al. Telemonitoring and self-management in the control of hypertension (TASMINH2): a randomised controlled trial. *Lancet* 2010; 376: 163-172.
3. Hoffmann-Petersen N, Lauritzen T, Bech JN, et al. Short-term telemedical home blood pressure monitoring does not improve blood pressure in uncomplicated hypertensive patients. J Hum Hypertens 2017; 31: 93-98.
4. McKinstry B, Hanley J, Wild S, et al. Telemonitoring based service redesign for the management of uncontrolled hypertension: multicentre randomised controlled trial. *BMJ* 2013; 346: f3030.
5. Bove AA, Homko CJ, Santamore WP, et al. Managing hypertension in urban underserved subjects using telemedicine - a clinical trial. *Am Heart J* 2013; 165: 615-621.
6. Margolis KL, Asche SE, Bergdall AR, et al. Effect of home blood pressure telemonitoring and pharmacist management on blood pressure control: a cluster randomized clinical trial. *JAMA* 2013; 310: 46-56.
7. McManus RJ, Mant J, Franssen M, et al. Efficacy of self-monitored blood pressure, with or without telemonitoring, for titration of antihypertensive medication (TASMINH4): an unmasked randomised controlled trial. *Lancet* 2018; 391: 949-959.
8. Bosworth HB, Powers BJ, Olsen MK, et al. Home blood pressure management and improved blood pressure control: results from a randomized controlled trial. *Arch Intern Med* 2011; 171: 1173-1180.
9. Maciejewski ML, Bosworth HB, Olsen MK, et al. Do the benefits of participation in a hypertension self-management trial persist after patients resume usual care? *Circ Cardiovasc Qual Outcomes* 2014; 7: 269-275.
10. Margolis KL, Asche SE, Dehmer SP, et al. Long-term outcomes of the effects of home blood pressure telemonitoring and pharmacist management on blood pressure among adults with uncontrolled hypertension: follow-up of a cluster randomized clinical trial. *JAMA Netw Open* 2018; 1: e181617.
11. Wakefield BJ, Koopman RJ, Keplinger LE, et al. Effect of home telemonitoring on glycemic and blood pressure control in primary care clinic patients with diabetes. *Telemed J E Health* 2014; 20: 199-205.
12. Logan AG, Irvine MJ, McIsaac WJ, et al. Effect of home blood pressure telemonitoring with self-care support on uncontrolled systolic hypertension in diabetics. *Hypertension* 2012; 60: 51-57.
13. Bray EP, Jones MI, Banting M, et al. Performance and persistence of a blood pressure self-management intervention: telemonitoring and self-management in hypertension (TASMINH2) trial. *J Hum Hypertens* 2015; 29: 436-441.
14. Kerry SM, Markus HS, Khong TK, et al. Home blood pressure monitoring with nurse-led telephone support among patients with hypertension and a history of stroke: a community-based randomized controlled trial. *CMAJ* 2013; 185: 23-31.
15. Fu SN, Dao MC, Luk W, et al. A cluster‐randomized study on the Risk Assessment and Management Program for home blood pressure monitoring in an older population with inadequate health literacy. *J Clin Hypertens (Greenwich)* 2020; 22: 1565-1576.
16. McManus RJ, Little P, Stuart B, et al. Home and online management and evaluation of blood pressure (HOME BP) using a digital intervention in poorly controlled hypertension: randomised controlled trial. *BMJ* 2021; 372: m4858.
17. Vedanthan R, Kamano JH, DeLong AK, et al. Community health workers improve linkage to hypertension care in western Kenya. *J Am Coll Cardiol* 2019; 74: 1897-1906.
18. Márquez Contreras E, Márquez Rivero S, Rodríguez García E, et al. Specific hypertension smartphone application to improve medication adherence in hypertension: a cluster-randomized trial. *Curr Med Res Opin* 2019; 35: 167-173.
19. Margolis KL, Bergdall AR, Crain AL, et al. Comparing pharmacist-led telehealth care and clinic-based care for uncontrolled high blood pressure: the Hyperlink 3 pragmatic cluster-randomized trial. *Hypertension* 2022; 79: 2708-2720.
20. Teo VH, Teo SH, Burkill SM, et al. Effects of technology-enabled blood pressure monitoring in primary care: A quasi-experimental trial. *J Telemed Telecare* 2024; 30: 121-130.
21. Levine DM, Dixon RF, Linder JA. Association of structured virtual visits for hypertension follow-up in primary care with blood pressure control and use of clinical services. *J Gen Intern Med* 2018; 33: 1862-1867.
22. Iljaž R, Brodnik A, Zrimec T, et al. E-healthcare for diabetes mellitus type 2 patients – a randomised controlled trial in Slovenia. *Zdr Varst* 2017; 56: 150-157.
23. Parsons SN, Luzio SD, Harvey JN, et al. Effect of structured self-monitoring of blood glucose, with and without additional telecare support, on overall glycaemic control in non-insulin treated type 2 diabetes: the SMBG study, a 12-month randomized controlled trial. *Diabet Med* 2019; 36: 578-590.
24. Fortmann AL, Gallo LC, Garcia MI, et al. Dulce digital: an mHealth SMS-based intervention improves glycemic control in Hispanics with type 2 diabetes. *Diabetes Care* 2017; 40: 1349-1355.
25. Naik AD, Hundt NE, Vaughan EM, et al. Effect of telephone-delivered collaborative goal setting and behavioral activation vs enhanced usual care for depression among adults with uncontrolled diabetes: a randomized clinical trial. *JAMA Netw Open* 2019; 2: e198634.
26. Odnoletkova I, Goderis G, Nobels F, et al. Optimizing diabetes control in people with type 2 diabetes through nurse-led telecoaching. *Diabet Med* 2016; 33: 777-785.
27. Yang Y, Lee EY, Kim H-S, et al. Effect of a mobile phone–based glucose-monitoring and feedback system for type 2 diabetes management in multiple primary care clinic settings: cluster randomized controlled trial. *JMIR Mhealth Uhealth* 2020; 8: e16266.
28. Warren R, Carlisle K, Mihala G, et al. Effects of telemonitoring on glycaemic control and healthcare costs in type 2 diabetes: a randomised controlled trial. *J Telemed Telecare* 2018; 24: 586-595.
29. Bujnowska-Fedak MM, Puchała E, Steciwko A. The impact of telehome care on health status and quality of life among patients with diabetes in a primary care setting in Poland. *Telemed J E Health* 2011; 17: 153-163.
30. Lim SL, Ong KW, Johal J, et al. Effect of a smartphone app on weight change and metabolic outcomes in Asian adults with type 2 diabetes. *JAMA Netw Open* 2021; 4: e2112417.
31. Liou JK, Soon MS, Chen CH, et al. Shared care combined with telecare improves glycemic control of diabetic patients in a rural underserved community. *Telemed J E Health* 2014; 20: 175-178.
32. Bohingamu Mudiyanselage S, Stevens J, Watts JJ, et al. Personalised telehealth intervention for chronic disease management: a pilot randomised controlled trial. *J Telemed Telecare* 2019; 25: 343-352.
33. Steventon A, Bardsley M, Doll H, et al. Effect of telehealth on glycaemic control: analysis of patients with type 2 diabetes in the whole systems demonstrator cluster randomised trial. *BMC Health Serv Res* 2014; 14: 334.
34. Tang PC, Overhage JM, Chan AS, et al. Online disease management of diabetes: engaging and motivating patients online with enhanced resources-diabetes (EMPOWER-D), a randomized controlled trial. *J Am Med Inform Assoc* 2013; 20: 526-534.
35. McFarland M, Davis K, Wallace J, et al. Use of home telehealth monitoring with active medication therapy management by clinical pharmacists in veterans with poorly controlled type 2 diabetes mellitus. *Pharmacotherapy* 2012; 32: 420-426.
36. Lee JY, Chan CKY, Chua SS, et al. Telemonitoring and team-based management of glycemic control on people with type 2 diabetes: a cluster-randomized controlled trial. *J Gen Intern Med* 2020; 35: 87-94.
37. Jia W, Zhang P, Zhu D, et al. Evaluation of an mHealth-enabled hierarchical diabetes management intervention in primary care in China (ROADMAP): a cluster randomized trial. *PLoS Med* 2021; 18: e1003754.
38. Anzaldo-Campos MC, Contreras S, Vargas-Ojeda A, et al. Dulce wireless tijuana: a randomized control trial evaluating the impact of project Dulce and short-term mobile technology on glycemic control in a family medicine clinic in northern Mexico. *Diabetes Technol Ther* 2016; 18: 240-251.
39. Vaughan EM, Hyman DJ, Naik AD, et al. A telehealth-supported, integrated care with CHWs, and medication-access (TIME) program for diabetes improves HbA1c: a randomized clinical trial. *J Gen Intern Med* 2021; 36: 455-463.
40. Christensen JR, Laursen DH, Lauridsen JT, et al. Reversing type 2 diabetes in a primary care-anchored eHealth lifestyle coaching programme in Denmark: a randomised controlled trial. *Nutrients* 2022; 14: 3424.
41. Lum ZK, Chang KL, Tsou KY, et al. Enhancing diabetes care with community pharmacist-involved collaborative care model: a multi-centre randomised controlled trial. *Diabetes Res Clin Pract* 2022; 185: 109238.
42. Gerber BS, Biggers A, Tilton JJ, et al. Mobile health intervention in patients with type 2 diabetes*. JAMA Netw Open* 2023; 6: e2333629.
43. Leong CM, Lee T-I, Chien Y-M, et al. Social media–delivered patient education to enhance self-management and attitudes of patients with type 2 diabetes during the COVID-19 pandemic: randomized controlled trial. *J Med Internet Res* 2022; 24: e31449.
44. Basudev N, Crosby-Nwaobi R, Thomas S, et al. A prospective randomized controlled study of a virtual clinic integrating primary and specialist care for patients with type 2 diabetes mellitus. *Diabet Med* 2016; 33: 768-776.
45. Benson GA, Sidebottom A, Hayes J, et al. Impact of ENHANCED (diEtitiaNs Helping pAtieNts CarE for Diabetes) telemedicine randomized controlled trial on diabetes optimal care outcomes in patients with type 2 diabetes. *J Acad Nutr Diet* 2019; 119: 585-598.
46. Choudhry NK, Isaac T, Lauffenburger JC, et al. Effect of a remotely delivered tailored multicomponent approach to enhance medication taking for patients with hyperlipidemia, hypertension, and dabetes: the STIC2IT cluster randomized clinical trial. *JAMA Intern Med* 2018; 178: 1182-1189.
47. Frias J, Virdi N, Raja P, et al. Effectiveness of digital medicines to improve clinical outcomes in patients with uncontrolled hypertension and type 2 diabetes: prospective, open-label, cluster-randomized pilot clinical trial. *J Med Internet Res* 2017; 19: e246.
48. Ramallo-Fariña Y, García-Bello MA, García-Pérez L, et al. Effectiveness of internet-based multicomponent interventions for patients and health care professionals to improve clinical outcomes in type 2 diabetes evaluated through the INDICA study: multiarm cluster randomized controlled trial. *JMIR Mhealth Uhealth* 2020; 8: e18922.
49. Stone RA, Rao RH, Sevick MA, et al. Active care management supported by home telemonitoring in veterans with type 2 diabetes: the DiaTel randomized controlled trial. *Diabetes Care* 2010; 33: 478-484.
50. Wild SH, Hanley J, Lewis SC, et al. Supported telemonitoring and glycemic control in people with type 2 diabetes: the Telescot diabetes pragmatic multicenter randomized controlled trial. *PLoS Med* 2016; 13: e1002098.
51. Nicolucci A, Cercone S, Chiriatti A, et al. A randomized trial on home telemonitoring for the management of metabolic and cardiovascular risk in patients with type 2 diabetes. *Diabetes Technol Ther* 2015; 17: 563-570.
52. Karhula T, Vuorinen AL, Rääpysjärvi K, et al. Telemonitoring and mobile phone-based health C-coaching among Finnish diabetic and heart disease patients: randomized controlled trial. *J Med Internet Res* 2015; 17: e153.
53. Wakefield BJ, Holman JE, Ray A, et al. Effectiveness of home telehealth in comorbid diabetes and hypertension: a randomized, controlled trial. *Telemed J E Health* 2011; 17: 254-261.
54. Weinstock RS, Teresi JA, Goland R, et al. Glycemic control and health disparities in older ethnically diverse underserved adults with diabetes: five-year results from the Informatics for Diabetes Education and Telemedicine (IDEATel) study. *Diabetes Care* 2011; 34: 274-279.

**APPENDIX 3: META-ANALYSES AND GRADE SCORES**

**CHANGE IN SBP RELATIVE TO STANDARD CARE**

**BP self-monitoring combined with teleconsultations in patients with AH after 6 months**

| **Study** | **Intervention** | | | **Standard care** | | |
| --- | --- | --- | --- | --- | --- | --- |
|  | **Mean** | **SD** | **Total** | **Mean** | **SD** | **Total** |
| **McManus et al. 2018** | -12.50 | 20.00 | 349 | -10.60 | 20.30 | 358 |
| **Kerry et al 2013** | +2.80 | 20.70 | 168 | +0.60 | 19.40 | 169 |
| **Fu et al 2020** | -12.00 | 19.60 | 152 | -2.00 | 17.20 | 137 |


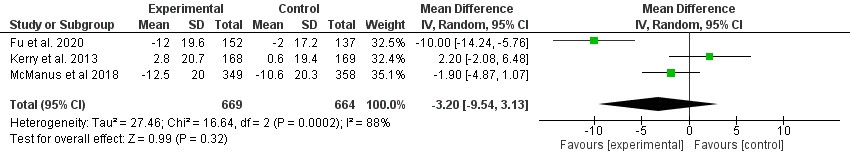


**GRADE SCORE**

| **Risk of bias** | **Inconsistency** | **Indirectness** | **Imprecision** | **Publication bias** | **Overall*** |
| --- | --- | --- | --- | --- | --- |
| Low | High | Low | High | Low | ⊕ ⊕ |

*Downgraded for inconsistency and imprecision.

**BP self-monitoring combined with teleconsultations in patients with AH after 12 months**

| **Study** | **Intervention** | | | **Standard care** | | |
| --- | --- | --- | --- | --- | --- | --- |
|  | **Mean** | **SD** | **Total** | **Mean** | **SD** | **Total** |
| **McManus et al.2018** | -15.90 | 20.30 | 328 | -12.70 | 21.00 | 358 |
| **Bray et al 2015** | -18.30 | 21.30 | 188 | -12.80 | 21.20 | 46 |
| **Kerry et al 2013** | +1.70 | 17.10 | 168 | -0.70 | 21.40 | 169 |
| **Fu et al 2020** | -21.00 | 17.50 | 152 | -10.00 | 17.20 | 137 |


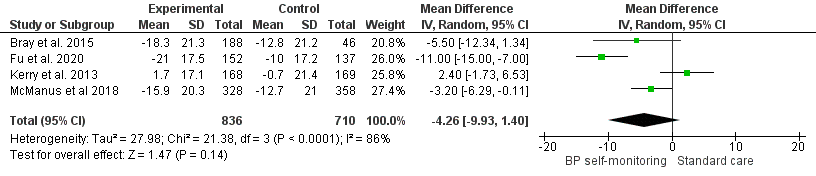


**GRADE SCORE**

| **Risk of bias** | **Inconsistency** | **Indirectness** | **Imprecision** | **Publication bias** | **Overall*** |
| --- | --- | --- | --- | --- | --- |
| Low | High | Low | High | Low | ⊕ ⊕ |

*Downgraded for inconsistency and imprecision.

**BP telemonitoring combined with teleconsultations in patients with AH after 6 months**

| **Study** | **Intervention** | | | **Standard care** | | |
| --- | --- | --- | --- | --- | --- | --- |
|  | **Mean** | **SD** | **Total** | **Mean** | **SD** | **Total** |
| **McManus et al. 2018** | -14.20 | 22.10 | 338 | -10.60 | 20.30 | 358 |
| **McKinstry et al. 2013** | -6.00 | 15.40 | 200 | -2.20 | 17.10 | 201 |
| **Bove et al 2013** | -18.20 | 20.30 | 120 | -13.90 | 18.20 | 121 |
| **Margolis et al 2013** | -21.50 | 17.47 | 206 | -10.80 | 17.79 | 197 |


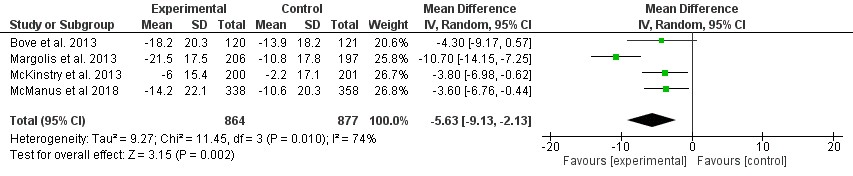


**GRADE SCORE**

| **Risk of bias** | **Inconsistency** | **Indirectness** | **Imprecision** | **Publication bias** | **Overall*** |
| --- | --- | --- | --- | --- | --- |
| Low | High | Low | Medium | Low | ⊕ ⊕ ⊕ |

*Downgraded for inconsistency.

**BP telemonitoring combined with teleconsultations in patients with comorbid AH and T2D after 6 months**

| **Study** | **Intervention** | | | **Standard care** | | |
| --- | --- | --- | --- | --- | --- | --- |
|  | **Mean** | **SD** | **Total** | **Mean** | **SD** | **Total** |
| **Stone et al. 2010** | -12.80 | 23.00 | 64 | -9.30 | 19.00 | 73 |
| **Nicolucci et al. 2015** | -6.70 | 8.90 | 153 | -7.30 | 11.10 | 149 |


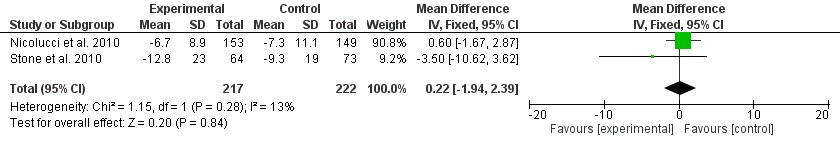


**GRADE SCORE**

| **Risk of bias** | **Inconsistency** | **Indirectness** | **Imprecision** | **Publication bias** | **Overall*** |
| --- | --- | --- | --- | --- | --- |
| Medium | Medium | Low | High | Uncertain | ⊕ ⊕ |

*Downgraded for imprecision and inconsistency.

**BP telemonitoring combined with teleconsultations in patients with AH after 12 months**

| **Study** | **Intervention** | | | **Standard care** | | |
| --- | --- | --- | --- | --- | --- | --- |
|  | **Mean** | **SD** | **Total** | **Mean** | **SD** | **Total** |
| **McManus et al. 2018** | -17.20 | 21.50 | 327 | -12.70 | 21.0 | 358 |
| **Margolis et al. 2013** | -22.50 | 18.50 | 197 | -12.85 | 18.57 | 191 |
| **Margolis et al 2022** | -18.70 | 28.80 | 1423 | -18.00 | 30.00 | 1648 |
| **Logan et al 2012** | -9.10 | 13.90 | 53 | -0.50 | 12.70 | 51 |


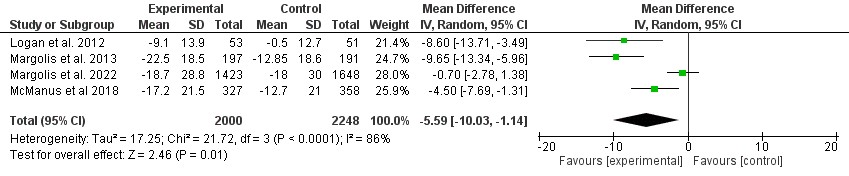


**GRADE SCORE**

| **Risk of bias** | **Inconsistency** | **Indirectness** | **Imprecision** | **Publication bias** | **Overall*** |
| --- | --- | --- | --- | --- | --- |
| Low | High | Low | Medium | Low | ⊕ ⊕ ⊕ |

*Downgraded for inconsistency.

**BP telemonitoring with teleconsultations in patients with comorbid AH and T2D after 12 months**

| **Study** | **Intervention** | | | **Standard care** | | |
| --- | --- | --- | --- | --- | --- | --- |
|  | **Mean** | **SD** | **Total** | **Mean** | **SD** | **Total** |
| **Nicolucci et al. 2015** | -6.70 | 8.50 | 153 | -7.60 | 11.10 | 149 |
| **Karhula et al 2015** | -6.10 | 18.50 | 148 | -4.10 | 12.80 | 60 |


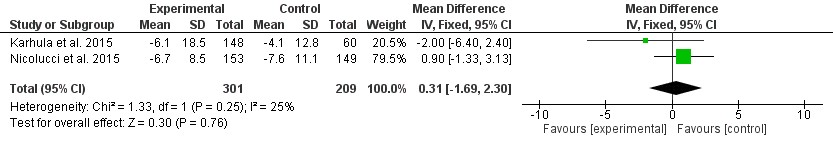


**GRADE SCORE**

| **Risk of bias** | **Inconsistency** | **Indirectness** | **Imprecision** | **Publication bias** | **Overall*** |
| --- | --- | --- | --- | --- | --- |
| Medium | Medium | Low | Medium | Uncertain | ⊕ ⊕ ⊕ |

*Downgraded for inconsistency.

**CHANGE IN HbA1c RELATIVE TO STANDARD CARE**

**BG self-monitoring combined with teleconsultations and tele-education in patients with T2D after 6 months**

| **Study** | **Intervention** | | | **Standard care** | | |
| --- | --- | --- | --- | --- | --- | --- |
|  | **Mean** | **SD** | **Total** | **Mean** | **SD** | **Total** |
| **Fortmann et al. 2017** | -1.00 | 1.20 | 50 | -0.20 | 1.70 | 69 |
| **Parsons et al. 2019** | -0.93 | 1.17 | 148 | -0.22 | 1.15 | 151 |
| **Odnoletkova et al. 2016** | -0.20 | 1.40 | 252 | 0.00 | 1.50 | 260 |
| **Liou et al. 2014** | -0.70 | 1.30 | 54 | -0.10 | 1.00 | 54 |
| **Vaughan et al. 2021** | -1.43 | 2.40 | 44 | -0.45 | 3.30 | 42 |
| **Lim et al. 2021** | -0.70 | 1.20 | 204 | -0.30 | 1.00 | 204 |
| **Iljaž et al. 2017** | -0.70 | 1.75 | 53 | -0.10 | 1.90 | 54 |


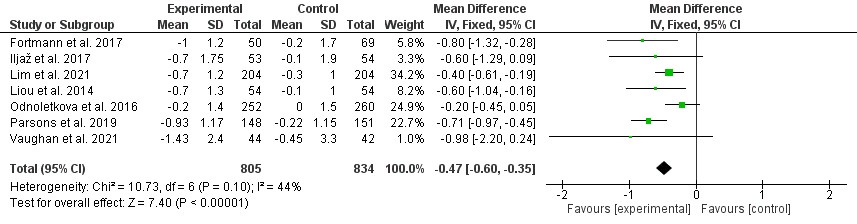


**GRADE SCORE**

| **Risk of bias** | **Inconsistency** | **Indirectness** | **Imprecision** | **Publication bias** | **Overall*** |
| --- | --- | --- | --- | --- | --- |
| Medium | Low | Low | Medium | High | ⊕ ⊕ ⊕ |

*Downgraded for publication bias.

**BG telemonitoring combined with teleconsultations in patients with T2D after 6 months**

| **Study** | **Intervention** | | | **Standard care** | | |
| --- | --- | --- | --- | --- | --- | --- |
|  | **Mean** | **SD** | **Total** | **Mean** | **SD** | **Total** |
| **Warren et al. 2018** | -0.90 | 0.90 | 63 | 0.00 | 1.10 | 63 |
| **Bujnowska Fedak et al. 2011** | -0.26 | 2.00 | 47 | -0.18 | 2.20 | 48 |
| **Tang et al. 2013** | -1.32 | 2.07 | 185 | -0.66 | 2.49 | 189 |
| **Lee et al. 2020** | -0.69 | 0.23 | 104 | -0.64 | 0.15 | 105 |
| **McFarland et al. 2012** | -2.10 | 1.70 | 67 | -1.60 | 1.20 | 36 |


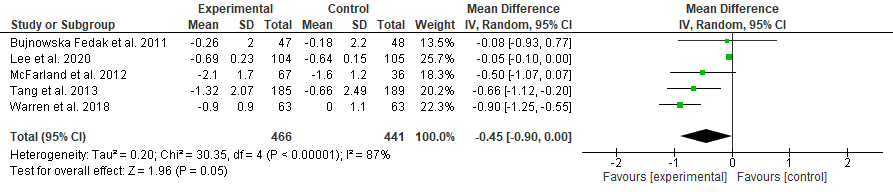


**GRADE SCORE**

| **Risk of bias** | **Inconsistency** | **Indirectness** | **Imprecision** | **Publication bias** | **Overall*** |
| --- | --- | --- | --- | --- | --- |
| Medium | Medium | Low | Medium | Low | ⊕ ⊕ ⊕ |

*Downgraded for imprecision.

**BG telemonitoring combined with teleconsultations in patients with comorbid T2D and AH after 6 months**

| **Study** | **Intervention** | | | **Standard care** | | |
| --- | --- | --- | --- | --- | --- | --- |
|  | **Mean** | **SD** | **Total** | **Mean** | **SD** | **Total** |
| **Stone et al. 2010** | -1.70 | 1.98 | 64 | -0.80 | 1.89 | 73 |
| **Nicolucci et al. 2015** | -0.62 | 1.13 | 153 | -0.29 | 1.20 | 149 |


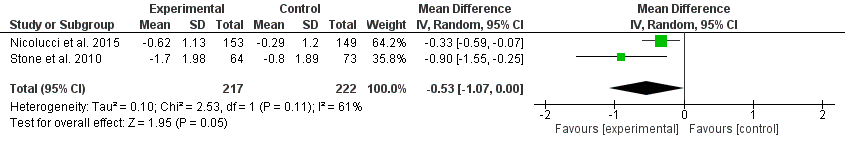


**GRADE SCORE**

| **Risk of bias** | **Inconsistency** | **Indirectness** | **Imprecision** | **Publication bias** | **Overall*** |
| --- | --- | --- | --- | --- | --- |
| Medium | Medium | Low | High | Uncertain | ⊕ ⊕ |

*Downgraded for imprecision and publication bias.

**BG telemonitoring combined with teleconsultations in patients with T2D after 12 months**

| **Study** | **Intervention** | | | **Standard care** | | |
| --- | --- | --- | --- | --- | --- | --- |
|  | **Mean** | **SD** | **Total** | **Mean** | **SD** | **Total** |
| **Steventon et al. 2014** | -0.23 | 2.24 | 266 | -0.03 | 2.28 | 191 |
| **Tang et al. 2013** | -1.14 | 2.29 | 186 | -0.95 | 2.44 | 193 |
| **Lee et al. 2020** | -0.33 | 0.21 | 104 | -0.30 | 0.15 | 104 |
| **Jia et al. 2021** | -0.33 | 1.75 | 11 731 | -0.01 | 1.78 | 5783 |


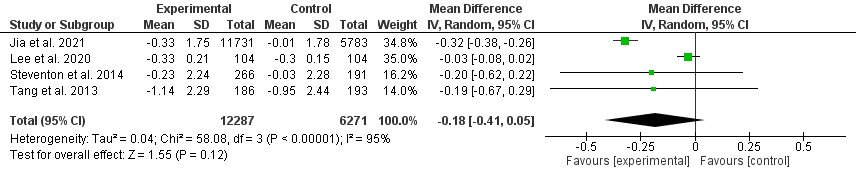


**GRADE SCORE**

| **Risk of bias** | **Inconsistency** | **Indirectness** | **Imprecision** | **Publication bias** | **Overall*** |
| --- | --- | --- | --- | --- | --- |
| Medium | High | Low | Medium | Low | ⊕ ⊕ ⊕ |

*Downgraded for inconsistency.

**BG telemonitoring with teleconsultations in patients with AH and T2D after 12 months**

| **Study** | **Intervention** | | | **Standard care** | | |
| --- | --- | --- | --- | --- | --- | --- |
|  | **Mean** | **SD** | **Total** | **Mean** | **SD** | **Total** |
| **Nicolucci et al. 2015** | -0.50 | 1.13 | 153 | -0.21 | 0.84 | 149 |
| **Karhula et al. 2015** | 0.04 | 0.82 | 156 | 0.18 | 0.72 | 61 |


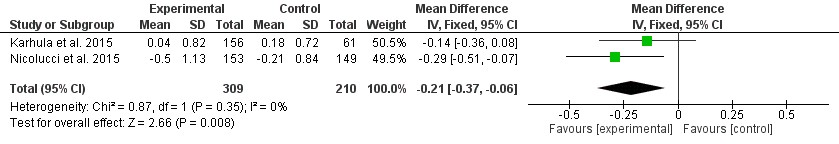


**GRADE SCORE**

| **Risk of bias** | **Inconsistency** | **Indirectness** | **Imprecision** | **Publication bias** | **Overall*** |
| --- | --- | --- | --- | --- | --- |
| Medium | Low | Low | Low | Uncertain | ⊕ ⊕ ⊕ |

*Downgraded for publication bias.

**Teleconsultations combined with tele-education in people with T2D after 6 months**

| **Study** | **Intervention** | | | **Standard care** | | |
| --- | --- | --- | --- | --- | --- | --- |
|  | **Mean** | **SD** | **Total** | **Mean** | **SD** | **Total** |
| **Lum et al. 2022** | -0.40 | 1.56 | 70 | -0.10 | 1.70 | 105 |
| **Naik et al. 2019** | -0.10 | 2.20 | 119 | -0.60 | 2.27 | 82 |
| **Christensen et al. 2022** | -0.76 | 1.15 | 75 | -0.61 | 0.72 | 37 |
| **Gerber et al. 2023** | -0.63 | 2.10 | 109 | -0.18 | 2.40 | 112 |


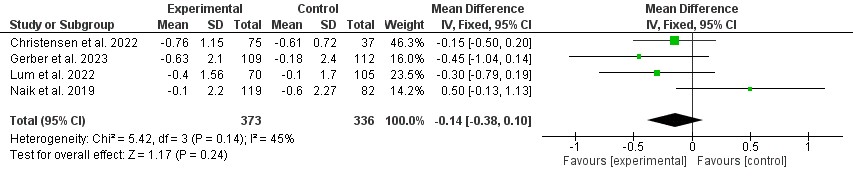


**GRADE SCORE**

| **Risk of bias** | **Inconsistency** | **Indirectness** | **Imprecision** | **Publication bias** | **Overall*** |
| --- | --- | --- | --- | --- | --- |
| Medium | Low | Low | High | Low | ⊕ ⊕ ⊕ |

*Downgraded for imprecision.

**Teleconsultations combined with tele-education in people with T2D after 12 months**

| **Study** | **Intervention** | | | **Standard care** | | |
| --- | --- | --- | --- | --- | --- | --- |
|  | **Mean** | **SD** | **Total** | **Mean** | **SD** | **Total** |
| **Naik et al. 2019** | -0.50 | 2.10 | 119 | -0.40 | 2.50 | **82** |
| **Gerber et al. 2023** | -0.81 | 2.08 | 84 | -0.19 | 2.43 | 99 |


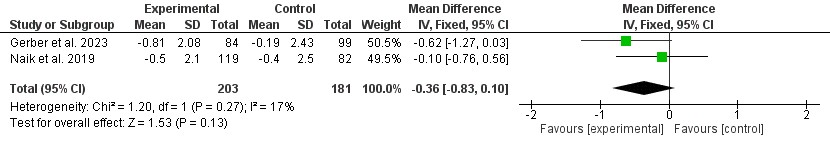


**GRADE SCORE**

| **Risk of bias** | **Inconsistency** | **Indirectness** | **Imprecision** | **Publication bias** | **Overall*** |
| --- | --- | --- | --- | --- | --- |
| Medium | Low | Low | High | Uncertain | ⊕ ⊕ |

*Downgraded for imprecision and publication bias.
